# Evaluating mechanical and optical patterns of translucent zirconia ceramics

**DOI:** 10.6026/973206300212811

**Published:** 2025-08-31

**Authors:** Neeraj Kumar Gupta, Sonal Tripathi, Swati Tripathi, Pramod Kumar Singhraul, Manan Shah, Ananya Bhargava, Miral Mehta

**Affiliations:** 1 Department of Dentistry, Shri Rawatpura sarkar institute of Medical sciences and research, Raipur, Chhattisgarh, India; 2 Department of Prosthodontics, Rishiraj College of Dental Sciences and Research Centre, Bhopal, Madhya Pradesh, India; 3 Department of Orthodontics and Orthopaedics, Rishiraj College of Dental Sciences and Research Centre, Bhopal Madhya Pradesh, India; 4 Department of Prosthodontics, New Horizon Dental College and Research Institute, Bilaspur, Chhattisgarh, India; 5 Department of Prosthodontics, Crown and Bridge, Faculty of Dental Science, Dharmsinh Desai University, Nadiad, Gujarat, India; 6 Department of Dentistry, Ruxmaniben Deepchand Gardi Medical College, Ujjain, Madhya Pradesh, India; 7 Department of Pediatric and Preventive Dentistry, Karnavati School of Dentistry, Karnavati University, Gandhinagar, Gujarat, India

**Keywords:** Translucent zirconia, flexural strength, translucency parameter, contrast ratio, cubic zirconia, 3Y-TZP, 5Y-PSZ

## Abstract

Translucent zirconia ceramics are increasingly used in dentistry, but improvements in translucency often compromise strength and alter microstructure. This *in vitro* study compared mechanical and optical properties of 3Y-TZP and 5Y-PSZ zirconia. Forty disc specimens were tested for biaxial flexural strength, surface roughness, translucency parameter (TP), contrast ratio (CR) and microstructure under SEM. Results showed 3Y-TZP had significantly higher flexural strength, whereas 5Y-PSZ exhibited superior translucency, with comparable surface roughness between groups. Thus, we show that 3Y-TZP is better suited for load-bearing regions, while 5Y-PSZ is preferable for anterior esthetic restorations, highlighting the need to balance strength and translucency in material selection.

## Background:

Zirconia-based ceramics have revolutionized the field of restorative dentistry owing to their superior mechanical properties, excellent biocompatibility and increasing aesthetic appeal [1]. Initially developed as an opaque material suited primarily for posterior restorations, zirconia ceramics have undergone significant improvements in translucency to meet the aesthetic demands of anterior restorations [2]. The introduction of high-translucency zirconia, particularly the cubic-containing variants such as 4Y-PSZ and 5Y-PSZ, has expanded the clinical applications of zirconia into regions traditionally dominated by glass ceramics. The mechanical performance of zirconia is primarily influenced by its phase composition and grain structure. Conventional 3Y-TZP (3 mol% yttria-stabilized tetragonal zirconia polycrystal) is characterized by a high flexural strength, largely due to its transformation toughening mechanism where the metastable tetragonal phase transforms into a monoclinic phase under stress, thereby resisting crack propagation [3]. However, to enhance translucency, newer formulations such as 5Y-PSZ (5 mol% yttria partially stabilized zirconia) incorporate a higher proportion of cubic phase, which lacks transformation toughening, thus reducing the material's mechanical resistance [4]. Optical properties such as translucency parameter (TP) and contrast ratio (CR) are critical for the esthetic integration of restorations, especially in anterior teeth [5]. These properties are significantly affected by factors like grain size, phase distribution and light scattering at grain boundaries. As the cubic phase increases, translucency improves due to lower birefringence and reduced light scattering. However, this comes at the cost of decreased flexural strength, necessitating a trade-off between esthetics and durability [6]. Several studies have attempted to characterize the optical and mechanical behavior of translucent zirconia materials using methods such as spectrophotometry, scanning electron microscopy (SEM) and biaxial flexural testing [7, 8- 9].

Yet, a comprehensive comparative evaluation of different translucent zirconia grades under standardized conditions remains limited. The evolution of zirconia ceramics into more translucent forms has led to significant modifications in their microstructural and optical behavior. The translucency is largely governed by the presence of the cubic phase, which has a lower refractive index mismatch and reduced birefringence compared to the tetragonal phase, thereby allowing greater light transmission. However, increasing the cubic content also results in diminished transformation toughening, which is the hallmark of 3Y-TZP zirconia's fracture resistance. Consequently, 5Y-PSZ, which contains approximately 50% cubic phase, offers excellent translucency but at the expense of reduced mechanical robustness, limiting its use to low-stress areas in the oral cavity [8]. Biaxial flexural strength is considered one of the most reliable parameters for evaluating the mechanical performance of ceramic materials because it replicates complex stress distributions similar to those in clinical conditions. Studies comparing 3Y-TZP and 5Y-PSZ have consistently reported that while 3Y-TZP can exceed 1000 MPa in strength, 5Y-PSZ typically falls in the range of 600-800 MPa, which is still clinically acceptable for anterior restorations. However, discrepancies in results often arise due to variations in fabrication techniques, sintering protocols and testing environments, making it necessary to assess materials under controlled, standardized laboratory conditions [10]. Optical performance, particularly the translucency parameter (TP), is quantitatively determined by measuring the color difference of a ceramic over white and black backgrounds using spectrophotometric analysis [11]. Therefore, it is of interest to report a comparative evaluation of the mechanical and optical properties of 3Y-TZP and 5Y-PSZ zirconia ceramics under standardized *in-vitro* conditions.

## Materials and Methods:

This *in-vitro* comparative study was conducted to evaluate the mechanical and optical characteristics of two types of translucent zirconia ceramics: 3 mol% yttria-stabilized tetragonal zirconia polycrystal (3Y-TZP) and 5 mol% yttria partially stabilized zirconia (5Y-PSZ). A total of 40 disc-shaped specimens (10 mm diameter x 1 mm thickness) were prepared, with 20 samples assigned to each group. The zirconia blocks was sectioned using a precision diamond saw under continuous water cooling to obtain uniform specimens. All samples were sintered according to the manufacturer's recommendations in a high-temperature furnace. Post-sintering, the specimens were polished with silicon carbide papers of increasing grit size (600, 800 and 1200) followed by alumina slurry for final finishing. Each sample was ultrasonically cleaned in distilled water for 10 minutes to remove surface debris.

## Mechanical testing:

Biaxial flexural strength was assessed using a universal testing machine (Instron®, USA). The samples were placed on a three-ball support fixture and loaded centrally at a crosshead speed of 1 mm/min until fracture. The maximum load at fracture was recorded and flexural strength was calculated using standard equations derived from ISO 6872 guidelines.

## Surface roughness:

Surface roughness (Ra) of each specimen was measured using a contact profilometer (Mitutoyo Surftest SJ-210). Three readings were taken per specimen at different locations and the average value was recorded in micrometers.

## Optical property evaluation:

Translucency parameter (TP) and contrast ratio (CR) were evaluated using a spectrophotometer (VITA Easyshade®) against standardized black and white backgrounds. Measurements were recorded in the CIE Lab* color space. TP was calculated as the color difference between readings on black and white substrates and CR was derived as the ratio of reflectance values on black versus white backgrounds.

## Microstructural analysis:

Selected specimens from each group were sputter-coated with gold and examined under a scanning electron microscope (SEM) at 5000x magnification to observe grain size and phase distribution. Grain size was calculated using ImageJ software by measuring a minimum of 100 grains per image. All experimental procedures were conducted under standardized laboratory conditions and data were subjected to statistical analysis using SPSS software (version 25.0). Independent t-tests were used to compare means between groups, with a significance level set at p < 0.05.

## Results:

The evaluation of mechanical and optical properties between 3Y-TZP and 5Y-PSZ zirconia ceramics demonstrated statistically significant differences in flexural strength and translucency, while surface roughness and contrast ratio showed less variation. Group A (3Y-TZP) exhibited higher mean biaxial flexural strength (987.3 ± 45.6 MPa) compared to Group B (5Y-PSZ) which showed lower values (723.4 ± 38.2 MPa). The difference was statistically significant (p < 0.01), indicating superior mechanical resistance in 3Y-TZP ceramics (Table 1 - see PDF). The surface roughness values showed minimal differences between groups. Group A had an average Ra of 0.21 ± 0.04 µm, while Group B recorded 0.23 ± 0.03 µm. The difference was not statistically significant (p = 0.17), suggesting that polishing and sintering protocols yielded similar surface textures (Table 2 - see PDF). Group B (5Y-PSZ) exhibited higher translucency (TP = 18.2 ± 1.4) than Group A (TP = 12.9 ± 1.1), reflecting improved optical transmission in the higher cubic content zirconia. The difference was statistically significant (p < 0.001) (Table 3 - see PDF). The contrast ratio values were slightly lower for 5Y-PSZ (CR = 0.74 ± 0.03) than for 3Y-TZP (CR = 0.81 ± 0.02), indicating greater translucency for the former. The differences were statistically significant (p < 0.05), though both materials maintained acceptable clinical ranges (Table 4 - see PDF). These results collectively indicate that 3Y-TZP provides better mechanical strength, while 5Y-PSZ offers superior optical performance, supporting their respective applications in high-stress and esthetic zones of dental restorations.

## Discussion:

The present study aimed to compare the mechanical and optical properties of two translucent zirconia ceramics, namely 3Y-TZP and 5Y-PSZ, which are widely used in prosthetic dentistry. The findings demonstrate a distinct trade-off between strength and translucency, consistent with earlier research that highlights compositional and structural differences between these materials [1, 2]. The higher biaxial flexural strength recorded in the 3Y-TZP group aligns with the well-documented transformation toughening mechanism inherent to the tetragonal phase [3]. The stress-induced transformation from the tetragonal to monoclinic phase improves crack resistance and plays a key role in the superior strength of 3Y-TZP ceramics [4]. contrast, the 5Y-PSZ samples showed significantly lower flexural strength, likely due to their higher cubic phase content, which lacks transformation toughening and leads to reduced fracture resistance [5, 6]. The findings regarding surface roughness indicate no significant differences between the two groups, suggesting that the polishing protocol and sintering conditions were adequate to produce comparable surface finishes. Previous studies have reported similar results, noting that surface roughness is influenced more by post-processing techniques than intrinsic material composition [7, 8]. Maintaining low surface roughness is essential for reducing plaque accumulation and enhancing the longevity of restorations [9]. The optical analysis revealed that 5Y-PSZ exhibited superior translucency, as evidenced by higher TP values and lower CR measurements. These findings are in line with earlier research that emphasizes the influence of cubic zirconia on light transmission due to its isotropic nature and lower light scattering [10, 11]. Increased grain size and reduced grain boundary density in cubic-rich compositions enhance translucency, making 5Y-PSZ ideal for esthetically demanding anterior restorations [12]. However, these optical advantages come at the expense of mechanical integrity, limiting their use in load-bearing areas [13]. Translucent zirconia ceramics offer a balance between strength and esthetics, showing greater translucency than conventional high-strength zirconia but lower than lithium disilicate, while maintaining superior mechanical resistance compared to lithium disilicate [14]. On the other hand, 5Y-PSZ samples showed larger grain sizes and a prominent cubic phase distribution, correlating with increased translucency but decreased strength. Microstructure, incorporation of a secondary phase, and sintering behavior can have a strong impact on the final mechanical and optical properties of dental ceramics [15]. In clinical practice, the selection of zirconia materials must be guided by the functional demands and esthetic requirements of the intended restoration site. For posterior teeth where occlusal forces are greater, 3Y-TZP remains the preferred material due to its superior strength. Conversely, in anterior regions where esthetics is prioritized, 5Y-PSZ offers a suitable balance with acceptable strength and enhanced optical behavior. The current findings reinforce the importance of understanding the interplay between mechanical and optical properties when choosing restorative materials.

## Conclusion:

3Y-TZP zirconia demonstrated superior mechanical strength, making it suitable for posterior load-bearing restorations, while 5Y-PSZ offered enhanced translucency ideal for anterior aesthetics. A balance between strength and esthetics is crucial in clinical material selection. Microstructural differences significantly influence both properties.
